# Comparative transcriptional profiling analysis of developing melon (*Cucumis melo* L.) fruit from climacteric and non-climacteric varieties

**DOI:** 10.1186/s12864-015-1649-3

**Published:** 2015-06-09

**Authors:** Montserrat Saladié, Joaquin Cañizares, Michael A. Phillips, Manuel Rodriguez-Concepcion, Christian Larrigaudière, Yves Gibon, Mark Stitt, John Edward Lunn, Jordi Garcia-Mas

**Affiliations:** IRTA, Centre for Research in Agricultural Genomics (CRAG), CSIC-IRTA-UAB-UB, Campus UAB, Bellaterra, Barcelona, 08193 Spain; COMAV, Institute for the Conservation and Breeding of Agricultural Biodiversity, Universitat Politècnica de València (UPV), Camino de Vera s/n, Valencia, 46022 Spain; Centre for Research in Agricultural Genomics (CRAG), CSIC-IRTA-UAB-UB, Campus UAB, Bellaterra, Barcelona, 08193 Spain; IRTA, Parc Científic i Tecnològic Agroalimentari, Parc de Gardeny, Edifici Fruitcentre, Lleida, 25003 Spain; Max Planck Institute of Molecular Plant Physiology, Wissenschaftspark Golm, Am Mühlenberg 1, Potsdam, 14476 (OT) Golm Germany; Present address: School of Chemistry and Biochemistry, Biochemistry and Molecular Biology, The University of Western Australia, Crawley, WA 6009 Australia; Present address: INRA Bordeaux, University of Bordeaux, UMR1332 Fruit Biology and Pathology, Villenave d’Ornon, F-33883 France

**Keywords:** Carotenoids, Fruit ripening, Ethylene, Fruit development, Microarray, Sucrose

## Abstract

**Background:**

In climacteric fruit-bearing species, the onset of fruit ripening is marked by a transient rise in respiration rate and autocatalytic ethylene production, followed by rapid deterioration in fruit quality. In non-climacteric species, there is no increase in respiration or ethylene production at the beginning or during fruit ripening. Melon is unusual in having climacteric and non-climacteric varieties, providing an interesting model system to compare both ripening types. Transcriptomic analysis of developing melon fruits from Védrantais and Dulce (climacteric) and Piel de sapo and PI 161375 (non-climacteric) varieties was performed to understand the molecular mechanisms that differentiate the two fruit ripening types.

**Results:**

Fruits were harvested at 15, 25, 35 days after pollination and at fruit maturity. Transcript profiling was performed using an oligo-based microarray with 75 K probes. Genes linked to characteristic traits of fruit ripening were differentially expressed between climacteric and non-climacteric types, as well as several transcription factor genes and genes encoding enzymes involved in sucrose catabolism. The expression patterns of some genes in PI 161375 fruits were either intermediate between. Piel de sapo and the climacteric varieties, or more similar to the latter. PI 161375 fruits also accumulated some carotenoids, a characteristic trait of climacteric varieties.

**Conclusions:**

Simultaneous changes in transcript abundance indicate that there is coordinated reprogramming of gene expression during fruit development and at the onset of ripening in both climacteric and non-climacteric fruits. The expression patterns of genes related to ethylene metabolism, carotenoid accumulation, cell wall integrity and transcriptional regulation varied between genotypes and was consistent with the differences in their fruit ripening characteristics. There were differences between climacteric and non-climacteric varieties in the expression of genes related to sugar metabolism suggesting that they may be potential determinants of sucrose content and post-harvest stability of sucrose levels in fruit. Several transcription factor genes were also identified that were differentially expressed in both types, implicating them in regulation of ripening behaviour. The intermediate nature of PI 161375 suggested that classification of melon fruit ripening behaviour into just two distinct types is an over-simplification, and that in reality there is a continuous spectrum of fruit ripening behaviour.

**Electronic supplementary material:**

The online version of this article (doi:10.1186/s12864-015-1649-3) contains supplementary material, which is available to authorized users.

## Background

Fruit-bearing plants can be broadly classified into two groups based on the type of fruit ripening: climacteric or non-climacteric [1]. In climacteric species, e.g. apple, banana, tomato and avocado, there is a sudden rise in respiration at the onset of ripening, which is accompanied by autocatalytic ethylene synthesis [2]. By contrast, there is little or no ethylene production in mature non-climacteric fruits, e.g. grape, *Citrus spp*, strawberry and pineapple [2]. However, there are many features of fruit ripening that are common to both climacteric and non-climacteric species indicating that there is overlap in the molecular mechanisms underlying the ripening process in both types, despite the differences in respiration and ethylene production. Ripening has a major impact on the organoleptic properties of the fruit, such as aroma, flavour, sweetness, acidity, colour and firmness [2–4]. Although the quality of some climacteric fruits generally deteriorates rapidly after peak maturity, limiting the potential market available to commercial fruit producers and providing suitable conditions for pathogen infection, there are some climacteric fruits that store for longer periods (e.g. apple). In contrast, some non-climacteric fruits tend to have a longer shelf life with fewer post-harvest losses during storage and transport, but non-climacteric fruits that are very perishable are also found (e.g. strawberry) [5, 6]. In nature, fruit ripening must be tightly coordinated with seed development to ensure that fruit consumption and seed dispersal by animals occurs only when the seeds are fully mature and viable. Thus there is both scientific and commercial interest in understanding the molecular mechanisms that differentiate climacteric from non-climacteric fruit ripening.

Climacteric fruit ripening has been extensively investigated in tomato (*Solanum lycopersicum*; syn*. Lycopersicon esculentum*) [7–9]. Most of the enzymes, receptors and other factors involved in ethylene synthesis, perception and signalling were first identified from analysis of ethylene response mutants in *Arabidopsis thaliana* [10], and orthologous proteins that are key components of the climacteric response have been found from comparative studies in tomato [11–13]. In addition, analysis of tomato mutants with impaired fruit ripening has revealed a number of transcription factors that are positive regulators of ripening including: RIPENING INHIBITOR [14], NON-RIPENING [15], COLORLESS NON-RIPENING [16], TOMATO AGAMOUS-LIKE-1 [17] and the homeobox protein HB-1 [18]. A member of the APETALA2 family has been identified as a negative regulator of ripening [19, 20]. Despite these advances, the molecular mechanisms involved in the initial triggering of the climacteric and the factors that determine the fruit’s responsiveness to exogenous ethylene remain largely unknown [21]. Furthermore, our knowledge of how metabolic changes and other aspects of fruit ripening are coordinated is fragmentary in both climacteric and non-climacteric species [22–25]. Only recently, epigenetic studies in tomato have suggested that epigenome modifications are important for the control of fruit ripening, and that they may work in concert with ethylene and fruit-specific transcription factors for the transition of fruit to the ripening competent stage [26].

Non-climacteric ripening has mostly been studied in strawberry, grape and *Citrus spp* [27–29]. However, comparison of these species with tomato has had limited success in elucidating the mechanisms that differentiate climacteric and non-climacteric ripening because of interspecific differences in gene/protein profiles and expression patterns that are unrelated to ripening. Integrative comparative analyses of transcript and metabolite levels have been performed in pepper and tomato during ripening [30] revealing new information about the metabolic regulation during climacteric and non-climacteric fruit development in these two closely related species. Melon is highly unusual in having both climacteric and non-climacteric varieties within the one species, making this an ideal subject to investigate the fundamental basis of the different ripening programs [31–33].

Climacteric melon varieties, such as the *reticulatus* and *cantalupensis* groups (e.g. cantaloupes), exhibit a characteristic burst of respiration and ethylene production at maturity [31, 34, 35]. This is usually accompanied by softening of the fruit, production of aromatic volatile compounds (e.g. esters, alcohols, apocarotenoids, sulphur-containing metabolites and aldehydes), fruit abscission, and ethylene-independent biosynthesis of β-carotene [4], which gives a characteristic orange colour to the flesh [36–39]. In contrast, non-climacteric melon varieties, such as the *inodorus* type [40, 41] do not undergo a respiratory burst or autocatalytic ethylene production. The fruits also have little aroma as they do not produce volatile esters, remain firm when ripe, are generally white-fleshed, and do not abscise from the mother plant. The lack of aroma or other obvious signs of ripeness make it more difficult for the grower to assess the optimal time for harvesting of these types, but this is offset by their longer shelf life.

To date, most investigations performed to improve knowledge of melon fruit ripening have been focussed on the analysis of climacteric types [42, 43]. A transcriptional analysis during fruit development and ripening in melon has been performed in the climacteric lines PI 414723 and Dulce [44], but the study was limited to genes associated with sugar accumulation [45] and the transcript data are not publicly available. A detailed transcriptomic analysis of the mature-fruit abscission zone in the Védrantais climacteric melon type has also recently been reported, identifying candidate genes associated with the induction of fruit abscission [46]. So far there are no studies describing transcriptomic changes during fruit development in non-climacteric fruit types.

The aim of the work presented here was to identify genes specific to climacteric or non-climacteric ripening by comparing patterns of gene expression during fruit development in melon varieties of the two types. Comparative transcriptomics in melon has been made possible by the development of a microarray with probes for over 17,000 melon unigenes [47] and sequencing of the melon genome [48]. We relate the observed changes in transcript abundance with changes in ethylene production, sugar levels, carotenoid content, and fruit firmness to identify candidate genes involved in determining these and other traits associated with fruit ripening.

## Results and discussion

### Climacteric and non-climacteric fruit types display distinct patterns of ethylene production and sugar and carotenoid accumulation

**Fig. 1:**
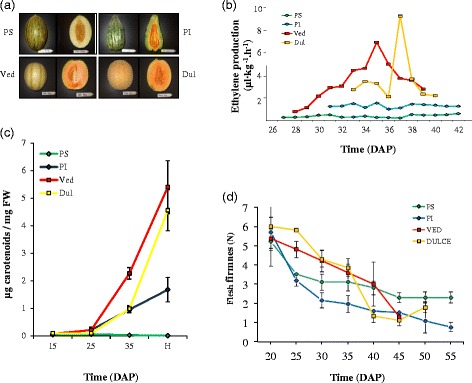
**a**: Ripe fruit of the four melon genotypes. The picture shows the differences in shape, size, external appearance (left) and flesh color (longitudinal cross, right). ‘Piel de Sapo’ (PS), PI 161375 (PI), ‘Dulce’ (Dul) and ‘Védrantais’ (Ved). **b**: Ethylene production. Endogenous ethylene production (μl · kg-1.h-1) of the four fruit genotypes at different time points. **c**: Carotene analysis. Total carotenoid content of melon fruit flesh of the four fruit genotypes at 15, 25, 35 DAP and harvest (H). **d**: Flesh firmness. Measured (in N) every 5 days until harvest for the four fruit genotypes

Two melon varieties, cv. Védrantais (Ved; *cantalupensis* group) and cv. Dulce (Dul; *reticulatus* group), were chosen as representatives of the climacteric type and compared with cv. Piel de sapo (PS; *inodorus* group) and accession PI 161375 (PI, *conomon* group) as non-climacteric types (Fig. 1a). Ved and Dul fruits showed a burst of ethylene production, peaking at 35 and 37 days after pollination (DAP), respectively, while PS and PI had lower and constant levels of ethylene production (Fig. 1b). When 1000 ppm of exogenous ethylene was applied to melons at the pre-ripe stage for 24 h at 20 °C, Ved and Dul fruits increased the production of endogenous ethylene, whereas PS and PI did not respond to the external treatment. We measured the expression of several ethylene related genes on PS, PI and Dulce, both in control and in ethylene treated fruits by qRT-PCR (Additional file 1: Figure S1). These results showed that the climacteric Dulce fruit increases the expression of ethylene inducible genes, compared with control fruits, when fruits where exogenously treated. However insensitive fruits, such as PS and PI, seem not to be affected by external ethylene (Additional file 1: Figure S1). These results confirmed the expected climacteric/non-climacteric behaviour of the four genotypes and suggest that the non-climacteric phenotypes of PS and PI may be due to impairment in ethylene production and/or sensing and downstream responses to exogenous ethylene.

Ripe Ved and Dul fruits have orange flesh, whereas the flesh of PS is white (Fig. 1a). PI has an outer region of green flesh and an inner core of orange flesh. Consistent with these visual phenotypes, the concentration of carotenoids increased in orange-fleshed melons during ripening (Ved, Dul and PI), whereas PS had very low carotenoid levels even in the mature fruits (Fig. 1c). In mature fruits, β-carotene was by far the most abundant carotenoid, with trace amounts detected even in the white-fleshed PS (Table 1). In addition to β-carotene, accession PI also accumulated substantial amounts of lutein, violaxanthin, β-cryptoxanthin and α-carotene (Table 1). Lutein and violaxanthin are accessory pigments in photosynthetic tissues, suggesting that these may have originated from the outer, green-fleshed regions of the PI fruit. For all genotypes there was a gradual decline in the firmness of the fruit during development, but in PS firmness remained constant from 45 DAP onwards (Fig. 1d).

**Table 1:** Carotenoid content of mature melon fruit from Védrantais (Ved; orange, harvested at 45 DAP), Piel de Sapo (PS; white, 55 DAP), Dulce (Dul; orange, 50 DAP) and PI 161375 (PI; green/orange, 50 DAP). Data are mean ± S.D. (*n* = 3). n.d. not detected

Carotenoid	Fruit carotenoid content (μg g^−1^FW)			
	Ved	PS	Dul	PI
Violaxanthin	n.d	n.d	n.d	129 ± 21
Lutein	n.d	n.d	19 ± 18	220 ± 28
β-cryptoxanthin	47 ± 13	n.d	24 ± 13	176 ± 47
α-carotene	42 ± 12	n.d	35 ± 3	153 ± 45
β-carotene	5316 ± 946	9 ± 3	4483 ± 946	998 ± 265

**Fig. 2:**
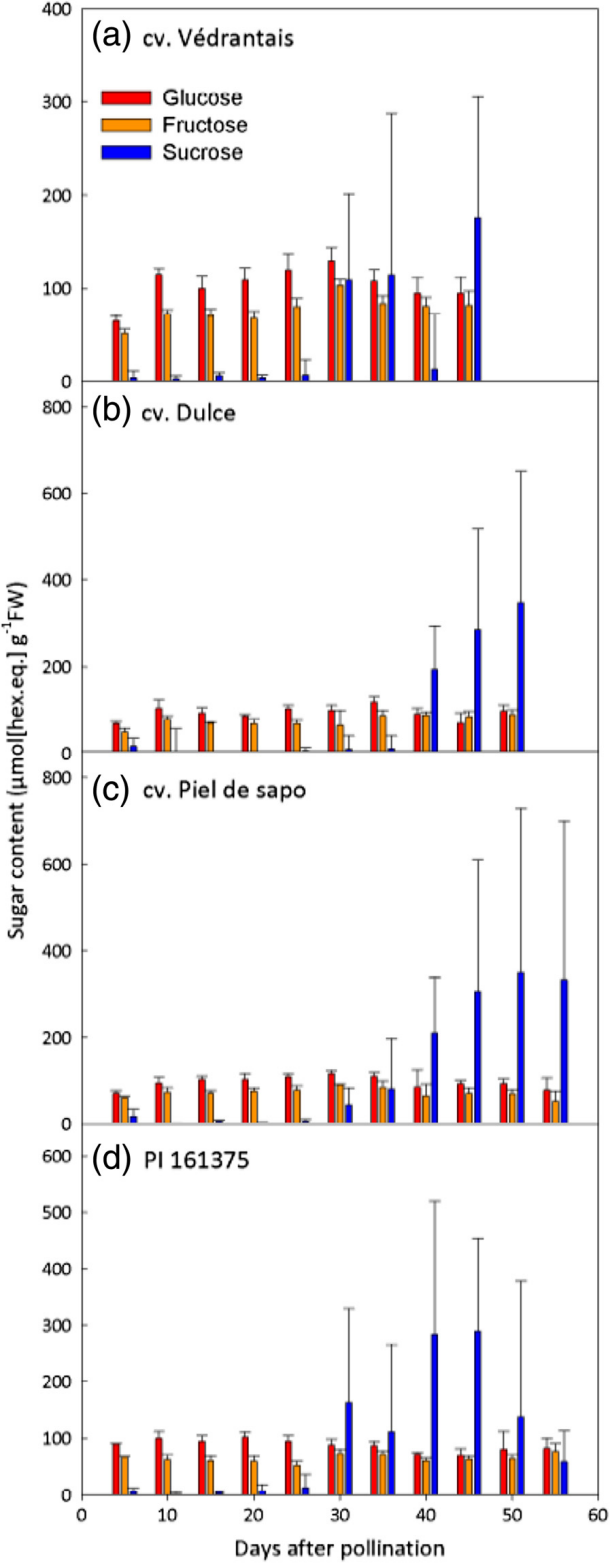
Soluble sugar content of melon fruits. Developing fruits were harvested from Ved, Dul, PS and PI at 5-day intervals from pollination until maturity, and the concentrations of glucose, fructose and sucrose at each sampling time were measured enzymatically

In climacteric melon varieties, fruit maturity is recognisable by the distinct aroma of the fruit, softening of the fruit, and often by the abscission of the fruit from the plant. In contrast, non-climacteric varieties do not show such obvious signs of ripeness, making comparison of genotypes at equivalent stages of development problematic. However, increasing sweetness is a feature common to some varieties from both climacteric and non-climacteric melons as they ripen and is one of the most important quality traits for the consumer. Therefore, we measured fruit sugar content at 5-day intervals to assess whether this could be used as an objective measure of ripeness for the non-climacteric varieties. Within each genotype there was little variation over time in the glucose and fructose content of the fruits between 5 days after pollination (DAP) and maturity at 55 DAP (PS and PI) or abscission of the fruit at 45–50 DAP (Ved and Dul) (Fig. 2). Sucrose levels were very low in young developing fruits (5–25 DAP) of all genotypes and then rose dramatically from 30 DAP (Ved, PS and PI) or 40 DAP (Dul) onwards (Fig. 2), suggesting that sucrose accumulation could be used as a marker for the onset of ripening. In all four genotypes there was considerable variation in sucrose content between individual fruits during the later stages of ripening, but the average sucrose content of PS fruits reached a plateau at 45 DAP, changing little thereafter up to 55 DAP. In contrast, PI fruits showed a clear drop in sucrose content by 55 DAP. In subsequent experiments, maturity was defined by abscission of the fruits for Ved (45 DAP) and Dul (50 DAP), while for PS (55 DAP) and PI (50 DAP) maturity was considered to be the last time point where the fruits had high sucrose content. Statistical analysis supporting differences in sugars accumulation is provided in Additional file 2: Table S1.

### Microarray analysis: microarray design, validation and global analysis

An oligo-based microarray was designed for transcriptomic analysis of melon fruits. The microarray contained 75,000 probes based on a previously validated microarray [47] but arranged in a modified 4-plex design, covering 17,443 melon unigenes derived from expressed sequence tags. The microarray has already been used to study the transcriptomic response during infection of melon with *Watermelon mosaic virus* [49] or *Monosporascus cannonballus* [50]. These unigenes represent 10,649 genes in the melon genome (38 % of the total predicted genes), with an additional 2021 unigenes with no assigned hit in the annotated melon genome (Additional file 3: Table S2). Triplicate samples of developing (15, 25 and 35 DAP) and mature fruits (as defined above) were collected for extraction of total RNA. After quality control checks, the RNA was reverse transcribed and the resulting cDNA hybridized to microarrays.

The microarray expression data were validated by real time quantitative reverse transcription PCR (qRT-PCR) of 12 gene transcripts in the four genotypes at each sampling time, using *CYCLOPHILIN7* (*CmCYP7*) as a reference gene (Table 2; Additional file 4: Table S3). From pairwise comparisons of microarray and qRT-PCR data, the mean (±SD) Pearson’s correlation coefficient for the 12 genes was 0.821 ± 0.191, indicating good overall agreement between the two methods (Additional file 5: Figure S2).

**Table 2:** Validation of microarray expression data by quantitative RT-PCR (qRT-PCR). Pearson correlation coefficients were calculated for microarray and qRT-PCR data for 12 gene transcripts. ICuGI indicates the International Cucurbit Genomics Initiative version 4.0 gene identifiers for each unigene (www.icugi.org)

Unigene Identifier	ICuGI	Gene name	Correlation
cCL451Contig1	MU46283	1-aminocyclopropane-1-carboxylate oxidase (*CmACO1*)	0.911
cPSI_11-E04-M13R_c	MU64933	N-acetyltransferase hookless1 (*CmACT1*)	0.896
cHS_07-B10-M13R_c	MU61400	DNAJ domain (*CmDNAj*)	0.510
cCL6170Contig1	MU54142	Ethylene-responsive transcriptional coactivator (*CmERT*)	0.943
cCI_54-H09-M13R	MU48499	Ethylene receptor (*CmETR1*)	0.371
cCL1086Contig1	MU43941	Expansin (*CmEXP1*)	0.724
cCL212Contig1	MU44581	NAM like protein (*CmNOR*)	0.931
cCL652Contig1	MU46792	ORANGE chaperone protein DNAJ-related (*CmORG*)	0.882
MU10927	MU50814	Polygalacturonase (*CmPG1*)	0.947
MU10942	MU49862	Phytoene synthase (*CmPSY1*)	0.871
cA_27-A08-M13R_c	MU51300	Terpenoid synthase (*CmTER*)	0.914
cCL6092Contig1	MU45836	Xyloglucan endotransglucosylase/hydrolase (*CmXTH1*)	0.962

**Fig. 3:**
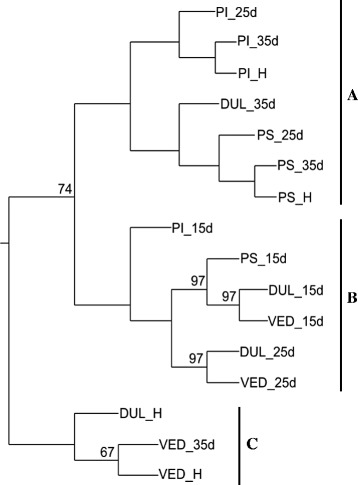
Hierarchical clustering analysis by UPGMA (Unweighted Pair Group Method with Arithmetic mean) with bootstrap, according to the differentially expressed genes, using the average of three biological replicates. PI (PI 161375), Dul (‘Dulce’), PS (‘Piel de Sapo’) and Ved (‘Védrantais’) at 15, 25, 35 DAP and H (harvest stage: 45 DAP for Ved, 50 DAP for Dul and PI, and 55 DAP for PS). Bootstrap values are only shown when lower than 100. **a**: ripe non climacteric fruits, **b**: unripe climacteric and non climacteric fruits and **c**: ripe climacteric fruits

Hierarchical clustering analysis of the normalized microarray data gave three main clusters (Fig. 3). Immature (15 DAP) fruits from all four genotypes were clustered together along with slightly older fruits (25 DAP) from the two climacteric varieties (Cluster B; Fig. 3). Fruits at intermediate stages of development (25–35 DAP) and mature fruits from PS and PI were clustered together with Dul fruits at 35 DAP (Cluster A; Fig. 3). The most divergent group (Cluster C; Fig. 3) included mature fruits from Ved and Dul, along with Ved fruits at 35 DAP, which coincided with the peak in ethylene production (Fig. 1b).

**Fig. 4:**
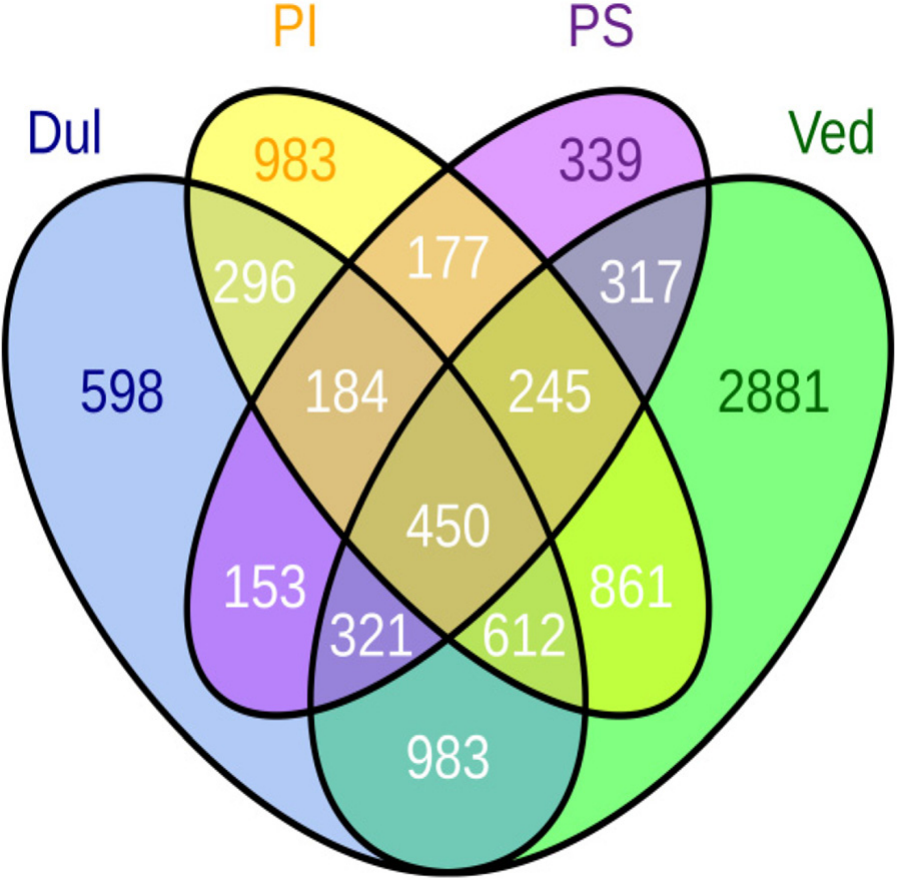
Venn diagram containing the differentially expressed genes shared by the four genotypes

Microarray Significant Profiles (MaSigPro) analysis [51] was conducted, with time as a continuous variable, to identify genes in each genotype that were differentially expressed at the four stages of development (≥2-fold difference between sampling times in at least one genotype). PS, PI, Ved and Dul showed 2186, 3808, 6670 and 3597 differentially expressed genes, respectively (Additional file 6: Table S4). Genes that were differentially expressed with time were compared across the four genotypes and classified into groups that were common to two, three or four genotypes, or were uniquely changed in only one genotype (Fig. 4). There were 450 genes that were differentially expressed over time in all four genotypes and a further 1362 genes in at least three genotypes (Fig. 4). Among the pairwise comparisons, there were 177 genes that were differentially expressed only in the non-climacteric genotypes (PS and PI), and a much larger number (983 genes) only in the climacteric varieties (Ved and Dul).

The differentially expressed genes were classified into Gene Ontology (GO) categories using FatiGO [52]. For most metabolic and developmental pathways there were no significant differences between genotypes for genes linked to those pathways. The main exceptions were the under-representation of genes associated with metabolism of nitrogen compounds and the over-representation of genes in the multicellular organism process category in PS compared with the other three genotypes (Additional file 7: Figure S3).

**Fig. 5:**
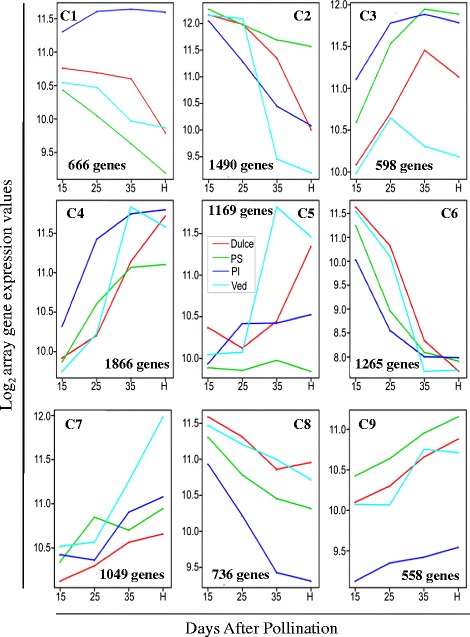
*K*-means clustering analysis of genes with different temporal expression profiles. Differentially expressed genes were separated in nine different clusters depending on their expression behaviour during fruit development and ripening. For each cluster, the number of differentially expressed genes is shown. Levels of gene expression are represented in the Y axis as raw fluorescence hybridization intensity data normalized by quantiles and transformed into log_2_ values, and fruit stage of development in the X axis. 15, 25, 35 are DAP (days after pollination) and H is the harvest stage (45 DAP for Ved, 50 DAP for Dul and PI, and 55 DAP for PS)

Further global analysis of the data was done by *k*-means clustering. This separated the 9400 differentially expressed genes into nine different clusters based on their temporal profiles during fruit development (Fig. 5). The genes in each cluster are listed in Additional file 8: Table S5. The corresponding GO terms from each cluster were analysed and the over-represented GO terms for each cluster are also shown in Additional file 8: Table S5. In cluster C1, genes showed decreasing expression throughout development in Ved, Dulce and PS, but not in PI. PI genes also showed weaker responses than the others in clusters C8 and C9. Expression of the genes in cluster C2 (Fig. 5) showed a strong downward trend as the fruits approached maturity in PI, Ved and Dul, with a weaker decrease in PS. Based on their GO terms, many of the 1490 genes in this cluster are related to photosynthesis and other plastid-related processes (Additional file 8: Table S5). Down-regulation of genes related to photosynthesis during fruit development has also been reported in grape [53]. The genes in cluster C5 (Fig. 5) showed a sharp rise in expression from 25 DAP in the two climacteric varieties, but were only marginally up-regulated or not at all in the two non-climacteric genotypes. Many of the 1169 genes in this cluster are related to biosynthetic processes and translation (Additional file 8: Table S5).

### Climacteric and non-climacteric genotypes differ in expression of ethylene biosynthesis and signalling related genes

**Fig. 6:**
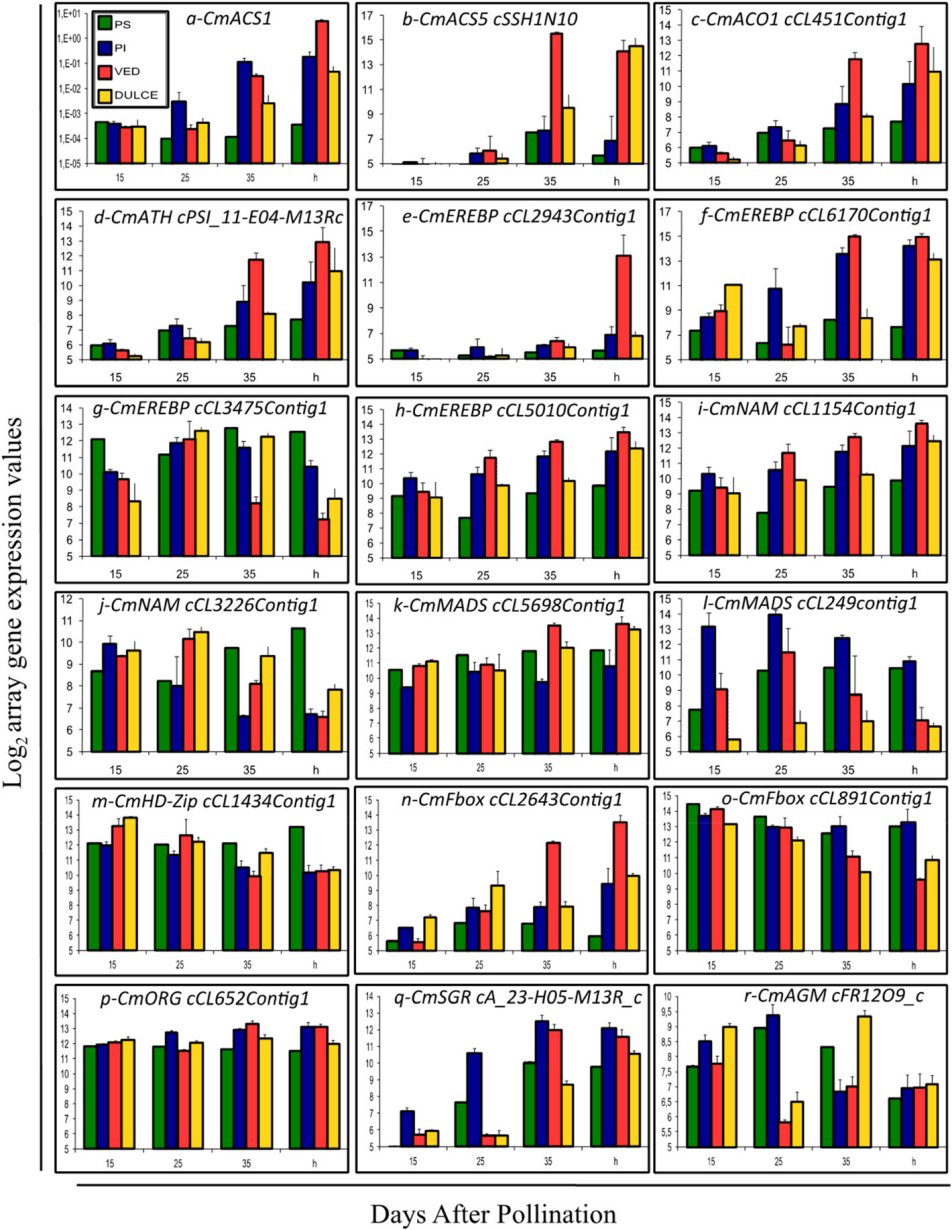
Expression of deregulated genes involved in the ethylene and carotenoid pathways. **a**: *CmACS1* (1-aminocyclopropane-1-carboxylate synthase 1), **b**: *CmACS5* (1-aminocyclopropane-1-carboxylate synthase 5:cSSH1N10), **c**: *CmACO1* (1-aminocyclopropane-1-carboxylate oxidase:cCL451Contig1), **d**: *CmATH* (N-acetyltransferase hookless1:cPSI_11-E04-M13R_c), **e-h**: *CmEREBP* (Ethylene-responsive element binding proteins: cCL2943Contig1, cCL6170Contig1, cCL3475Contig1 and cCL5010Contig1), **i-j**: *CmNAM* (Non Apical Meristem/NAC domain proteins: cCL1154Contig1 and cCL3226Contig1), **k-l**: *CmMADS* (MADS-box transcription factor: cCL5689Contig1 and cCL2496Contig1), **m**: *CmHD-Zip* (BZIP transcription factor: cCL1434Contig1), **n-o**: *CmF-box* (F-box transcription factor:cCL2643Contig1 and cCL891Contig1), **p**: *CmORG* (Orange gene, chaperone protein dnaJ-like protein:cCL652Contig1), **q**: *CmSGR* (STAY-GREEN2 protein:cA_23-H05-M13R_c) and **r**: CmAGM (Agamous-like MADS-box protein:cFR12O9_c)

Ethylene is synthesized from S-adenosyl methionine in a two-step pathway catalyzed by 1-aminocyclopropane-1-carboxylate synthase (ACS; EC 4.4.1.14) and 1-aminocyclopropane-1-carboxylate oxidase (ACO; EC 1.14.17.4). The melon genome contains eight *ACS* and five *ACO* genes [48]. Four *ACS* genes were represented on the melon microarray: *CmACS2*, *CmACS3*, *CmACS4* and *CmACS5* (Additional file 9: Table S6). *CmACS1* is known to be fruit specific and ripening related as its expression increases in climacteric fruit after the burst of ethylene [54, 55]. As this gene was not present on the microarray its expression was measured by qRT-PCR. Expression of *CmACS1* increased up to 10,000-fold during ripening not only in the two climacteric varieties (Ved and Dul) but also in non-climacteric PI (Fig. 6a). From the microarray analysis, it was observed that *CmACS5*, a previously uncharacterized member of the melon *ACS* gene family, was strongly induced in Ved and Dul fruits, coinciding with the climacteric burst of ethylene production (Fig. 6b and Additional file 10: Table S7). Only two ESTs for *CmACS5* are present in the International Cucurbit Genomics Initiative (ICuGI; http://www.icugi.org) database, both from fruit tissue from climacteric melon types, suggesting that it is specifically expressed in climacteric fruit types.

Four out of the five melon *ACO* genes annotated in the melon genome were represented on the microarray: *CmACO1*, *CmACO2*, *CmACO3* and *CmACO5. CmACO1* expression increased during ripening in both the climacteric varieties and in PI, but was relatively constant throughout fruit development in PS (Fig. 6c; Additional file 10: Table S7). The changes in *CmACO1* expression were also confirmed by qRT-PCR (Additional file 5: Figure S2).

The expression of genes related to ethylene perception and signalling was also analysed. Three ethylene receptors have been identified in the melon genome (*CmETR1-CmETR3*). *CmETR1* and *CmETR2* are represented on the microarray and their transcripts detected, but neither showed substantial differences in expression between genotypes or sampling times (Additional file 10: Table S7). Similarly, expression of the melon *ETHYLENE-INSENSITIVE3*-*LIKE* transcription factor genes (*CmEIL1* and *CmEIL2*) was constant throughout fruit development and ripening in all genotypes (Additional file 10: Table S7). A putative *N-ACETYLTRANSFERASE HOOKLESS1 g*ene (*CmATH*), similar to an ethylene response gene from *Arabidopsis thaliana* [56], was strongly induced during ripening in climacteric fruits and PI, but showed no significant change in expression in PS during ripening (Fig. 6d; Additional file 10: Table S7).

There are several classes of transcriptional regulators implicated in downstream ethylene signalling pathways, such as trans-acting ethylene-responsive element binding proteins (EREBP). Fifty-four melon unigenes with homology to known *EREBP* genes were represented on the microarray, and expression of some of these differed greatly between the melon genotypes during fruit development and ripening. Expression of the melon *EREBP-*like unigenes cCL2943Contig1, cCL6170Contig1, cCL5010Contig1, cCI_03-A07-M13R_c and cCL1517Contig2 increased during ripening in all genotypes except PS (Fig. 6e-f, h; Additional file 10: Table S7). Two other related unigenes, c15d_17-C05-M13R_c and cCL3475Contig1, were constantly and highly expressed in PS, but down-regulated in climacteric fruits concomitantly with the burst of ethylene production (Fig. 6g; Additional file 10: Table S7).

Other gene families involved in ethylene regulation and signalling during ripening encode several classes of transcription factors as MADS, NAM/NAC, F-box and HD-Zip [14, 15, 18, 57, 58], and differentially expressed genes belonging to these classes are discussed below in a specific section dedicated to transcription factors.

### Differential expression of carotenoid related genes

Genes encoding enzymes or regulators of carotenoid biosynthesis were identified from the melon genome sequence [48] and those represented on the melon microarray are listed in Additional file 10: Table S7. A *CmGGH* gene (cCL1327Contig1) encoding a putative geranylgeranyl reductase (EC 1.3.1.83) was much more strongly expressed in the genotypes with orange (Ved and Dul) or green/orange (PI) fruit flesh than in the white-fleshed PS (Additional file 10: Table S7). This enzyme is involved in the biosynthesis of the phytyl moiety of chlorophyll [59], but might also be involved in salvaging the phytyl moiety when chlorophyll is degraded, producing geranylgeranyl diphosphate, which is the precursor of carotenoid biosynthesis. Other genes encoding enzymes of carotenoid biosynthesis did not show major differences in expression between the orange or orange/green and white-fleshed genotypes. However, a lycopene epsilon cyclase (cCL4493Contig1; EC 5.5.1.18) gene was strongly induced in PI from 25 DAP to maturity but not in the other three genotypes (Additional file 10: Table S7). This enzyme catalyses the initial reaction in the main pathway of lutein synthesis via α-carotene [60], so increased expression of lycopene epsilon cyclase might account for the higher α-carotene and lutein contents of mature PI fruit compared to the other genotypes (Table 1).

A number of transcription factors have been associated with regulation of carotenoid biosynthesis in other species, and nine melon homologs of these were represented on the microarray (Additional file 10: Table S7). Expression of the tomato *APETALA2* gene was reported to be negatively correlated with carotenoid accumulation [19], and a similar correlation was seen for its homolog in melon, *CmEREBPF* (cCL3475Contig1) (Additional file 10: Table S7). Another ethylene responsive transcription factor gene, *CmEREBP*/*RAP2-3* (cCL5010Contig1) was more highly expressed in orange-fleshed melon fruits than in PS (Fig. 6h; Additional file 10: Table S7) and showed a positive correlation with carotenoid accumulation like its RAP2-2 homolog in *Arabidopsis thaliana* [61]. Expression of a leucine zipper homeobox protein gene, *CmHD-ZIP* (cPSI_41-F06-M13R_c), increased during ripening in orange-fleshed melons but not in PS (Additional file 10: Table S7). Repression of the homologous tomato gene (*LeHB1*/*HD-ZIP*) inhibited ripening and carotenoid accumulation in the fruits [18].

Carotenoid accumulation can be also affected by changes at the whole-plastid level. Chloroplastic heat shock proteins (HSP) affect the conversion of chloroplasts to non-photosynthetic chromoplasts, which in turn affects the pigment profile of the plastids [62]. Expression of *CmORG* (cCL652Contig1), which is orthologous with a DnaJ-like protein encoding gene in the *orange* (*org*) mutant of cauliflower, was relatively constant in PS, but increased from 25 DAP in the orange-fleshed melons, especially Ved (Fig. 6p; Additional file 10: Table S7). Accumulation of β-carotene in the cauliflower *org* mutant is associated with a metabolic process that triggers the differentiation of plastids into chromoplasts rather than changes in expression of genes encoding carotenoid biosynthetic enzymes [63, 64]. The *CmORG* gene maps to a region of the melon genome containing the white flesh colour (*wf*) locus [38], and thus is a strong candidate gene for this locus.

The tomato *stay green* (*sgr*) mutant is unable to degrade chlorophyll and chlorophyll-binding proteins during senescence, leading to retention of chlorophyll and has effects on fruit ripening [65]. A melon ortholog of the tomato *STAY GREEN* gene, *CmSGR* (cA_23_H05-M13Rc), was more strongly induced during ripening in orange or orange/green flesh than in white-fleshed PS (Fig. 6q; Additional file 10: Table S7). Over-expression of tomato *AGAMOUS-LIKE1* (*TAGL1*), a MADS box transcription factor, decreased carotenoid and ethylene levels, suppressed chlorophyll breakdown, and down-regulated expression of ripening-associated genes in the fruit [17]. Expression of a melon *AGAMOUS* (*CmAGM*; cFR1209_c) homolog was higher in PS and PI than Ved and Dulce at 25 DAP, consistent with the lower carotenoid content of the former two varieties, but the expression pattern of this gene shifted at later time points and its expression was similar in all four genotypes at maturity (Fig. 6r; Additional file 10: Table S7).

### Differential gene expression between Vedrantais and Piel de Sapo

Although many genes showed differential expression in the two climacteric varieties compared to non-climacteric PS, the expression patterns in PI were intermediate, suggesting that PI shows some characteristics of climacteric varieties, even though it does not display a classical climacteric burst of ethylene production (Fig. 1b). The near isogenic line SC3-5-1, which contains two introgressions of PI in the background of PS, shows a climacteric ripening phenotype [33], indicating that PI alleles of QTLs *ETHQB3.5* and *ETHQV6.3* are capable of inducing ethylene production in PS, and that the absence of ethylene production in PI may be due to mutations in other genes. As the inclusion of this genotype in the comparative transcriptomic analysis might obscure some of the differences between climacteric and non-climacteric varieties, we analysed differentially expressed genes between only one variety of each ripening type, Ved and PS, to see if this might reveal more clearly genes that are specifically associated with climacteric or non-climacteric ripening. These two varieties are representative of the two major types of melon in commercial production for the fresh fruit market. The Ved and PS microarray datasets were compared using the Significance Analysis of Microarrays (SAM) tool in the TM4 software package [66] and differentially expressed genes are listed in Additional file 11: Table S8. The data were also analysed using Robin software [67] and differentially expressed genes were categorised using MapMan ontology to identify which processes are likely to be transcriptionally regulated during ripening of climacteric and non-climacteric fruits [68, 69].

#### Ethylene biosynthesis and signalling

**Fig. 7:**
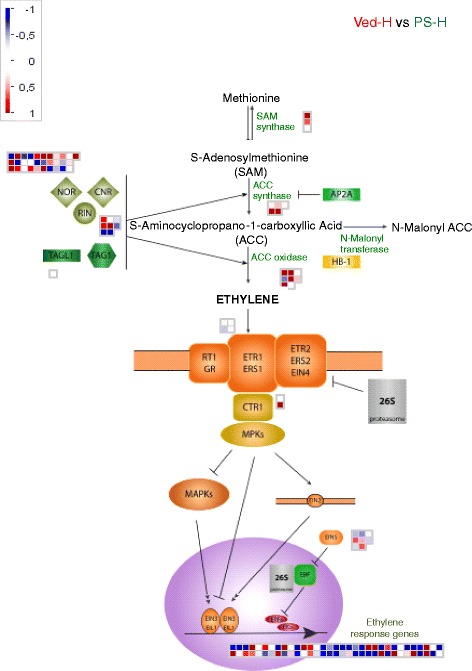
Differential expression of ethylene biosynthesis related genes in mature Ved and PS fruits. Differences in gene expression were visualised using MapMan [68]. The red squares represent genes that were more highly expressed in Ved and the blue squares the genes that were more highly expressed in PS. The genes and their expression levels are listed in Additional file 11: Table S8

**Fig. 8:**
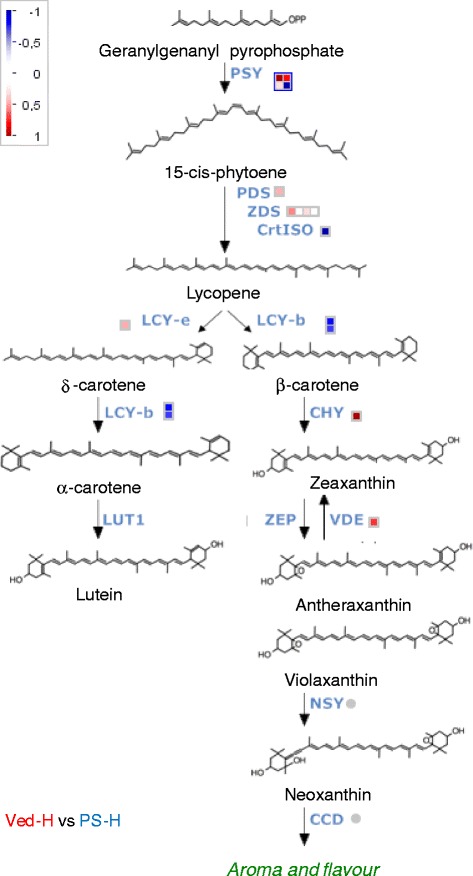
Differential expression of carotenoid biosynthesis related genes in mature Ved and PS fruits. Differences in gene expression were visualised using MapMan [68]. The red squares represent genes that were more highly expressed in Ved and the blue squares the genes that were more highly expressed in PS. The genes and their expression levels are listed in Additional file 11: Table S8. PSY: phytoene synthase; PDS: phytoene desaturase; ZDS: *ζ-*carotene desaturase; CrtISO: carotenoid isomerase; LCY-e: lycopene *ε*-cyclase; LCY-b: lycopene *β*-cyclase; CHY: carotenoid *β*-hydroxylase; LUT1: carotenoid *ε*-hydroxylase; ZEP: zeaxanthin epoxidase; VDE: violaxanthin de-epoxidase; NSY: neoxanthin synthase; CCD: carotenoid cleavage dioxygenase

**Fig. 9:**
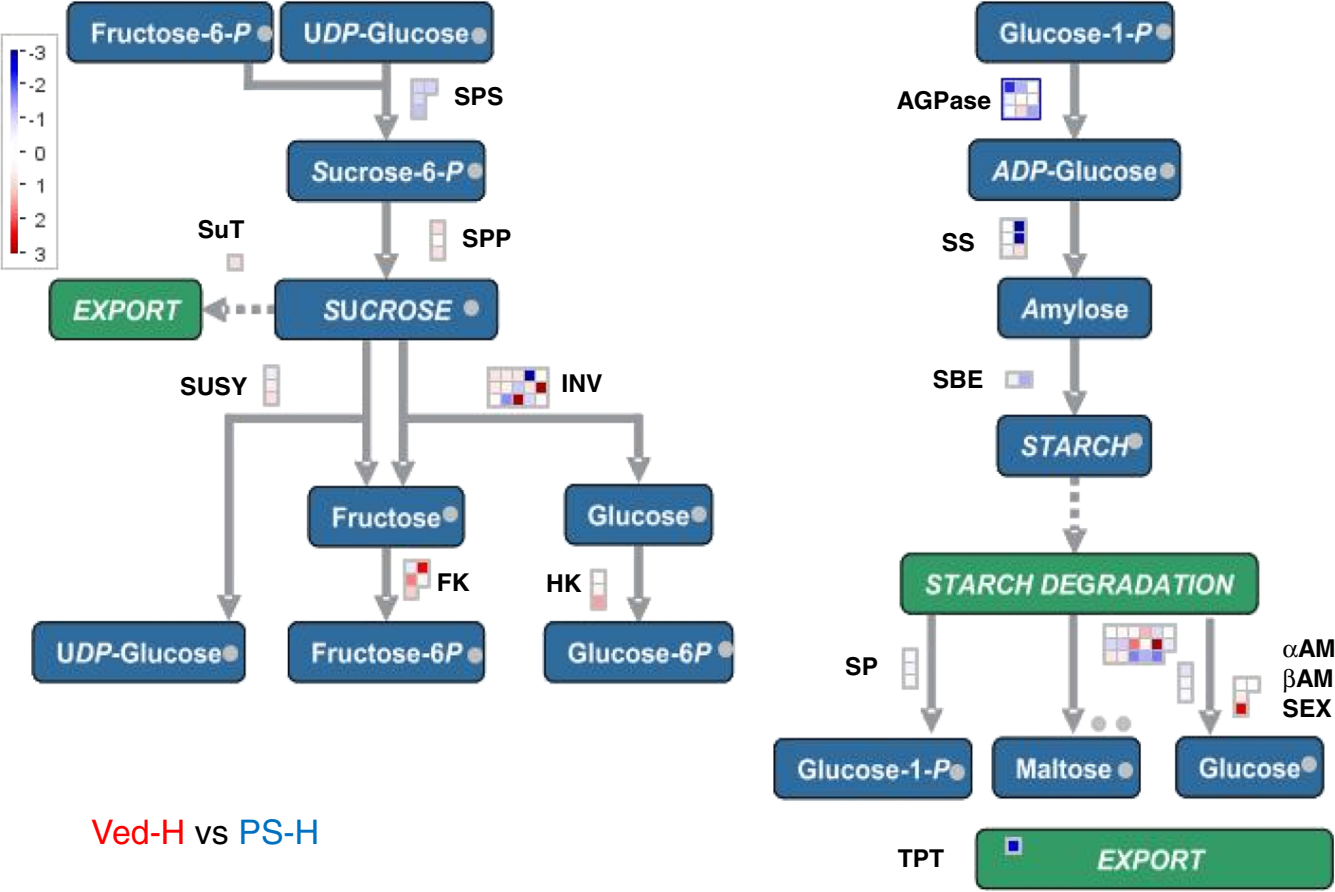
Differential expression of sugar metabolism related genes in mature Ved and PS fruits. Differences in gene expression were visualised using MapMan [68]. The red squares represent genes that were more highly expressed in Ved and the blue squares the genes that were more highly expressed in PS. The genes and their expression levels are listed in Additional file 11: Table S8. SPS: sucrose-P-synthase; SPP: sucrose-P-phosphatase; INV: invertase; SUSY: sucrose synthase; SuT: sucrose transporter; FK: fructokinase; HK: hexokinase; AGPase: sucrose synthase; SS: starch synthase; SBE: starch branching enzyme; SP: starch phosphorylase; *α*AM: *α*-amylase; *β*AM: *β*-amylase; SEX: water dikinase; TPT: triose phosphate transporter

MapMan displays of genes that are differentially expressed in mature Ved and PS fruits are shown in Figs. 7, 8 and 9. The red squares represent genes that were more highly expressed in Ved and the blue squares the genes that were more highly expressed in PS. The genes and their expression levels are listed in Additional file 11: Table S8. In the pathway of ethylene biosynthesis (Fig. 7), *CmSAM3* (encoding S-adenosylmethionine synthetase 3; c15d_41-c10-m13r_c), *CmACS5* (cSSH1N10_c) and *CmACO1* (cCL451Contig1) were more highly expressed in Ved than in PS (see also Additional file 11: Table S8). Similarly, two genes for downstream components of the ethylene signalling pathway, *CmATH* (cPSI_11-E04-M13R_c) and *CmERT* (cCL6170Contig1) were 3 to 5-fold more highly expressed in Ved than PS. The *CmRAP2-3* gene (cCL5010Contig1), encoding an EREBP ethylene-responsive transcription factor was also more highly expressed in Ved, whereas related ethylene-responsive element-binding factors such as c15d_17-C05-M13R_c and cCL3475Contig1 unigenes were more highly expressed in PS.

#### Carotenoid biosynthesis and photosynthesis related genes

Among genes associated with carotenoid biosynthesis, *CmCHYE/Lut1* (cCI_04-E08-M13R_c; encoding a carotene ɛ-hydroxylase) and *CmPSY1* (MU10942 and cSSH9H13_c; encoding a phytoene synthase) were more highly expressed in Ved than PS, whereas *CmPSY3* (cHS_38-F04-M13R_c), *CmCRTISO* (cCL1236Contig1; encoding a carotenoid isomerase) and *CmZEP* (cCL3831Contig1; encoding a zeaxanthin epoxidase) were preferentially expressed in PS (Fig. 8). Carotenoids are synthesized from the isoprenoid precursor geranylgeranyl pyrophosphate (GGPP), and the *CmGGPS* gene (cHS_10-E06-M13R_c; geranylgeranyl pyrophosphate synthase) was expressed at a higher level in Ved than PS fruits, as was the *CmGGH* (cCL1327Contig1; geranylgeranyl reductase) involved in metabolism of GGPP and the phytyl moiety of chlorophyll (Additional file 11: Table S8). Genes encoding proteins involved in light harvesting and the photosynthetic electron transport chain were strongly expressed in both genotypes at 15 DAP. During ripening, there was a large decrease in expression of photosystem II related genes in Ved so that at maturity their expression was much higher in PS than Ved (data not shown). However, some chlorophyll a-b binding protein and ATP synthase (γ and δ subunits) genes showed the opposite pattern (data not shown).

#### Transcription factors

A total of 1,044 transcription factor genes were represented on the microarray and 985 of these were differentially expressed at different stages of development. At maturity, 134 transcription factor genes were more highly expressed in Ved fruits than in PS, while 181 showed the opposite pattern (Additional file 11: Table S8). Among the differentially expressed transcription factor genes, the most common classes were NAC/NAM, MYB, AP2 domain, bZIP, MADS-box, bHLH and SBP-box proteins, which are also the main types of transcription factors that are differentially expressed during tomato fruit ripening [70].

A total of 27 fruit-expressed unigenes encoding NAM/NAC-domain proteins were represented on the array. There was very wide variation in expression of these genes between genotypes and developmental stages. Of particular interest was a NAC domain transcription factor gene, *CmNAC/JA* (cCL1154Contig1), which is homologous with the tomato *NOR* gene [15]. *CmNAC/JA* was expressed at a lower level in PS than in Ved and Dul at harvest. In contrast, the expression of the NAC-domain unigene cCL3226Contig1 increased during ripening in PS but decreased in PI, Ved and Dul (Fig. 6i-j; Additional file 10: Table S7 and Additional file 11: Table S8).

The MADS box protein RIPENING-INHIBITOR (RIN) was proposed to function early in the transcriptional activation cascade regulating ripening-related processes, including ethylene production and/or signalling [14]. Seven MADS-box related genes were represented on the microarray. Two of these genes, *CmMADS8* (cCL2496Contig1) and cCL5689Contig1, were specifically down-regulated and up-regulated in the climacteric genotypes, respectively (Fig. 6k-l; Additional file 10: Table S7 and Additional file 11: Table S8).

An HB-1 homeobox protein transcription factor (HD-ZIP) was recently reported to regulate fruit ripening in tomato [18] by binding to the *SlACO1* promoter, thereby activating this gene’s expression and therefore fruit ripening. The melon unigene cCL1434Contig1 has homology with HD-ZIP transcription factors. Its expression strongly declined during development of climacteric fruits, showed a smaller decrease in PI but increased during ripening in PS (Fig. 6m). The expression of other transcription factor genes from this family increased during ripening in climacteric fruits, but not in PS (Additional file 10: Table S7 and Additional file 11: Table S8).

F-box proteins are involved in regulation of key proteins involved in ethylene signalling by targeting them for degradation via the polyubiquitin-26S proteasomal pathway. In tomato, silencing of the F-box genes *Sl-EBF1* and *Sl-EBF2* resulted in a constitutive ethylene response phenotype, an accelerated plant senescence and earlier fruit ripening, suggesting that they are integral components of ethylene-dependent developmental processes [58]. The role of these F-box proteins in tomato ripening would be to target EIN3 for degradation, thus down-regulating ethylene signalling. Several F-box protein genes were found to be differentially expressed between genotypes during fruit development and ripening (Additional file 10: Table S7). Among these, unigene cCL2643Contig1 was strongly induced in climacteric fruits, and to a lesser extent in PI, during ripening, but was expressed at a lower level and decreased during ripening in PS (Fig. 6n). In contrast, expression of unigene cCL891Contig1 was relatively constant in the non-climacteric genotypes but decreased after the burst of ethylene production in Ved and Dul (Fig. 6o).

There was also differential expression of EREBP, MYB and WRKY protein genes and transcription factor genes associated with auxin signalling and the ubiquitin pathway, but different members of these gene families showed opposite trends, with some more highly expressed in Ved while others were preferentially expressed in PS (Additional file 11: Table S8).

#### Sugar and cell wall metabolism

Fruits of all four melon genotypes accumulated high levels of sucrose as they ripened (Fig. 2). Sucrose is stored primarily in the vacuoles of the pericarp parenchyma cells and is the major determinant of fruit sweetness, which is one of the most important organoleptic traits for the consumer. Melon, in common with other cucurbits, is a symplastic phloem loading species that exports raffinose and stachyose as well as sucrose from source leaves to sink tissues, such as developing fruits [71]. Imported raffinose family oligosaccharides are hydrolysed by α-galactosidases, releasing sucrose and galactose. The latter is phosphorylated by galactokinase (GK) and the resulting galactose 1-phosphate converted to other hexose-phosphates, providing the substrates for synthesis of sucrose by sucrose-phosphate synthase (SPS) and sucrose-phosphate phosphatase (SPP). There are multiple genes encoding α-galactosidase in melon [45] and several were highly expressed in developing melon fruits (cSSH9H24_c, cA_15-C06-M13R_c or cSSH9I1_c), although the predominantly expressed α-galactosidase gene differed between Ved and PS (Additional file 11: Table S8). There were only minor differences in expression of *GK*, *SPS* and *SPP* genes between the two varieties (data not shown).

Sucrose can be catabolised by sucrose synthase or invertase. Expression of a *CmINV* gene (cCL4861contig1), encoding a soluble (vacuolar) acid invertase, was almost 10 times higher in Ved than PS (Fig. 9; Additional file 11: Table S8). In contrast, one of the *CmCWINV* genes (c46d_34-C03-M13R_c), encoding a cell wall invertase, was preferentially expressed in PS. Expression of two *CmINVINH* genes (cCL2226Contig1 and c15d_02-B02-M13R_c), encoding invertase inhibitor proteins, was about 30 times higher in PS than Ved. The highly expressed vacuolar invertase in Ved might limit the accumulation of sucrose during ripening, and contribute to rapid post-harvest decline in sucrose levels. Catabolism of sucrose by invertase will not only diminish the sweetness of the fruit, but also feed sugars into respiratory pathways that produce organic acids, such as malate, that impart a stale flavour to the fruit. Even though one of the *CmCWINV* genes was highly expressed in ripe PS fruits, cell wall invertase might not have access to intracellular (vacuolar) stores of sucrose. The high expression of invertase inhibitors might be expected to inhibit the invertase. These invertase and invertase inhibitor expression patterns could be an important factor in maintaining high sucrose levels in PS at maturity and also limit post-harvest losses of sucrose, thus contributing to the long shelf life of this variety.

The overall firmness of melon fruits decreases as they ripen in non-climacteric as well as climacteric varieties (Fig. 1d), although localized softening at the proximal end of the fruit is particularly pronounced in climacteric types. Genes encoding polygalacturonases (*CmPG*), glucan endo-1,3-β-glucosidases (*CmGLU*) and β-d-xylosidases (*CmXYL*) are known to be induced by ethylene [43, 72] and several of these were more strongly up-regulated during ripening in Ved than PS (e.g. cCL3465, MU10927, cFR14J7_c, cCL3761Contig1, cCL1814Contig1; Additional file 11: Table S8). Increased expression and activity of these enzymes is expected to promote cell wall degradation and softening of the fruit. However, other cell wall related genes (e.g. a fascilin-like arabinogalactanan protein gene, *CmFAG* cCL1228Contig1) were more highly expressed in PS than in Ved, suggesting that loss of fruit firmness in PS is mediated by a different set of enzymes and is probably independent of ethylene signalling.

#### Other differentially expressed genes

When the SAM analysis was performed, a total of 65 unigenes showed a ≥2-fold higher expression in Ved than in PS and 137 unigenes showed a ≥2-fold higher expression in PS than in Ved (Additional file 11: Table S8, spreadsheet 2). Apart from genes already described before, genes that were notably up-regulated in Ved compared to PS included: two UDP-glucosyltransferases cCI_13-F09-M13R_c and cPSI_24-F06-M13R_c (polysaccharide synthesis), an anthocyanin 5-aromatic acyltransferase cCI_35-A04-M13R_c (anthocyanin modification), a β-1,3-glucanase-like protein cCL3761Contig1 (callose degradation), an l-allo-threonine aldolase cCL2708Contig1 (glycine synthesis) and a bidirectional sugar transporter cA_19-H06-M13R_c. Apart from genes already described before, genes that were more strongly expressed in PS than Ved included: a cytochrome P450-like protein c15d_31-D11-M13R_c (monooxygenase/oxidation of organic substances), HSF30 cSSH6A3_c (heat-shock protein), a deoxyhypusine synthase cCL449Contig1 (polyamine metabolism/modification of eukaryotic translation initiation factor 5A), F-box proteins c15d_35-A07-M13R_c and cPS_21-E08-M13R_c, and several proteins related to auxin or gibberellin signalling. Auxin signalling is known to be involved in the initiation of ripening in the non-climacteric strawberry fruit [73].

## Conclusions

There is massive re-programming of transcription during fruit development and ripening in climacteric and non-climacteric melon varieties, with many gene expression changes common to both types. However, major differences in gene expression were observed in the climacteric varieties, Ved and Dul, when compared with non-climacteric PS, especially genes related to ethylene biosynthesis and signalling. Changes in flesh colour during ripening appear to be linked to conversion of chlorophyll containing chloroplasts into carotenoid containing chromoplasts, including chlorophyll degradation and recycling of the phytyl moieties, consistent with previous reports [39]. An up-regulation of genes involved in the synthesis of isoprenoid precursors of carotenoids during ripening of orange-fleshed Ved melon, but not the white-fleshed PS, was recently reported [74]. By contrast, only minor changes were observed here in genes encoding the enzymes involved in the synthesis of the carotenoids themselves.

Many transcription factors showed differential expression when climacteric and non-climacteric fruits where compared, suggesting that transcriptional regulatory networks differ between varieties and contribute to differences in fruit ripening behaviour. Neither PS nor PI exhibited a burst of ethylene production during fruit ripening, and so were classified as non-climacteric varieties. However, other features of PI fruits, such as the high carotenoid content and induction of some ethylene biosynthetic genes, were more similar to the climacteric varieties Ved and Dul. In many respects, the transcript profile of PI represented a midpoint between the climacteric and non-climacteric genotypes. This intermediate behaviour suggests that ripening behaviour in melon should be considered as a continuous spectrum with classical non-climacteric varieties such as PS at one end and climacteric varieties such as Ved and Dul at the other. This suggests that climacteric ripening is not determined by a single gene but by the interactions of multiple genes, in agreement with the identification of multiple loci affecting ethylene biosynthesis in several mapping populations [33, 75]. Sucrose content can be used as a marker of melon fruit ripeness, even in non-climacteric varieties that show no visible or other signs of ripeness. The sucrose content of mature fruit and post-harvest losses of sucrose might be influenced by up-regulation of a specific soluble (vacuolar) acid invertase in climacteric melon fruits, while expression of two invertase inhibitors could explain the high and stable levels of sucrose in non-climacteric melon fruits, and thus be an important factor in their longer shelf-life.

## Methods

### Plant material

Melon (*Cucumis melo* L.) plants were grown under the same greenhouse conditions as described in [76]. Flowers (one per plant) were hand pollinated to precisely determine the stage of development, and fruits were harvested at 15, 25 and 35 DAP and at maturity as determined by fruit abscission in Ved (45 DAP) and Dulce (50 DAP), or by the maximal sucrose content in PI (50 DAP) and PS (55 DAP). For each genotype and sampling time, flesh (mesocarp) was collected from three separate fruits. Samples were taken from the middle of the fruit, avoiding rind, seed and jelly tissues, immediately frozen in liquid nitrogen and stored at −80 °C until analysis. After cutting the fruit in half without removing the rind, flesh firmness was measured from the inner side using a hand penetrometer fitted with an 8-mm cylindrical probe (Fruit pressure tester, model FT-011; Italy).

### Analysis of ethylene production

Three fruits of PS (31 DAP), PI (27 DAP), Ved (28 DAP) and Dul (33 DAP) were harvested at the time indicated in parentheses and kept at 20 °C. Ethylene production was measured daily for 16 days by sealing individual fruits of known weight for 1 h in 5-L plastic jars fitted with sampling septa. The ethylene concentration in 1-mL samples of the headspace of each jar was measured with a Thermo Finnigan gas chromatograph (Trace GC 2000, Milan, Italy) equipped with a flame ionization detector. For experiments involving external ethylene treatment of fruits, plants were cultivated in a greenhouse under the same conditions described above. Pre-climacteric mature fruits (Ved 27 DAP, Dul 35 DAP, PS 49 DAP and PI 37 DAP) were harvested and treated immediately at 20 °C for 24 h with 1000 ppm of ethylene gas, injected by syringe into chambers to achieve desired ethylene concentrations. A 25 ml solution of 1 M KOH was also placed inside the chambers to maintain low concentrations of CO_2_ from respiration. Control melon fruits were exposed to air under the same conditions. To measure endogenous ethylene production fruits where ventilated for 3 h after the external treatment and then sealed individually in fresh 5-L plastic jars for another hour. This was done to ensure that the ethylene measured would be the one produce by the fruit. Ethylene was measured as described before. After the treatment, fruits were placed 1 day at room temperature and then the mesocarp tissue was frozen in liquid nitrogen and stored at −80 °C for subsequent gene expression analysis.

### Measurement of sugars and carotenoids

Sucrose, glucose and fructose were assayed enzymatically in ethanolic extracts as in [77]. Total carotenoids were extracted from frozen melon fruit flesh and saponified as described in [78] with the following modifications: the dried pellet containing the carotenoids was resuspended in 50 μL acetone:CHCl_3_ (1:1) just prior to HPLC analysis, the samples were shielded from heat and light following homogenization, and canthaxanthin (50 ng · mg^−1^ FW of tissue) was added to the frozen material prior to homogenization as an internal standard. Individual carotenoids (violaxanthin, lutein, β-cryptoxanthin, α-carotene and β-carotene) were separated by reverse-phase HPLC on C30 column attached to a Waters Alliance 2690 chromatography system equipped with a diode array detector operating from 200–500 nm [79]. Quantification of β-carotene in melon extracts was based on comparison to an external standard curve using serial dilutions of an authentic standard. Recovery of the canthaxanthin internal standards was estimated by comparison to its standard curve, and the concentration of β-carotene in the fruit samples corrected accordingly. Additional carotenoid peaks were identified by comparing their spectral properties and retention times under similar chromatographic conditions as reported in the literature [80–82].

### RNA isolation and synthesis of double stranded cDNA

Total RNA from three different biological replicates for each stage and genotype was isolated from mesocarp tissue using a Plant RNeasy® Mini Kit (Qiagen, Hilden, Germany). RNA was treated with RNAse free TURBO-DNase I (Turbo-DNA-*free*^TM^ Kit; Applied Biosystems, Ambion®, USA) for 30 min at 37 °C, before use as a template for cDNA synthesis. RNA quality was assessed by agarose gel electrophoresis, spectophotometric analysis with a Nanodrop ND-1000 (NanoDrop® Technologies, Wilmington, Delaware) and analysis on an Agilent 2100 Bioanalyzer (Agilent Technologies, Palo Alto, CA). High-quality RNA samples were reversed transcribed into cDNA using a SuperScript™ double-stranded cDNA synthesis kit (Invitrogen, Carlsbad, CA). The integrity of cDNA was assessed as described above for RNA. A minimum of 2.5 μg of cDNA per sample was supplied for labelling and hybridization on custom-made melon microarrays.

### Design and hybridization of the melon NimbleGen® microarray

A basic 4-plex melon oligo-based gene expression microarray was designed in collaboration with Roche NimbleGen, based on a previously validated probe set derived from 33,000 melon expressed sequence tags (ESTs) from 12 normalized cDNA libraries, which were obtained from different melon tissues from plants grown under different physiological conditions [47]. In addition, 244 new unigenes were represented on the microarray, 33 from the GenBank database (http://ncbi.nlm.nih.gov) and 209 ESTs from the International Cucurbit Genomics Initiative database (ICuGI; http://www.icugi.org/). In total, 17,443 melon unigenes were represented on the custom microarray, with four 60-mer oligo nucleotides per unigene, making a total of 75,000 probes [49, 50]. The microarray was estimated to cover 38 % of all the genes in the melon genome, with a potentially higher proportion of fruit-expressed genes due to over-representation of fruit tissue in the original cDNA libraries.

Hybridization of melon fruit cDNA samples (three biological replicates per genotype and treatment) to the custom microarray was performed by NimbleGen® following their own specifications (NimbleGen Arrays User’s Guide; http://www.nimblegen.com).

### Microarray data analysis

Raw data were analysed without background subtraction using the oligo package from Bioconductor (http://www.bioconductor.org). Background was corrected by the robust multi-array average (RMA) method. The data were normalized by quantiles, model-fitted using Tukey’s Median Polish method and transformed into log_2_ values. Differentially expressed genes were extracted using the Bioconductor Microarray Significant Profiles (MaSigPro) R package for the analysis of single and multiseries time course microarray experiments [51]. MaSigPro follows a two step regression strategy to find genes with significant temporal expression changes and significant differences between experimental groups. The method defined a general regression model for the data. For our experiments with four time points a cubic regression model (degree = 3) was defined. First, the global model was adjusted by the least-squared technique to identify differentially expressed genes, and significance was assessed by applying a false discovery rate (Q = 0.01). Second, a variable selection procedure was applied to find significant variables for each gene, for which a stepwise regression was employed (step.method = “two.ways.backward”, α = 0.01). Then, lists of differentially expressed genes according to each variable were generated (rsq = 0.6). After the MaSigPro analysis, a cut-offoflog_2_ (−fold difference) ≥1 (i.e. at least two-fold difference) was applied to designate genes as differentially expressed. Genes whose expression did not change were excluded to minimize background noise.

Hierarchical clustering was performed using Euclidean distance by UPGMA (Unweighted Pair Group Method with Arithmetic mean) with bootstrapping (100 replicates). Gene clustering was also performed using the *k*-means method [83] with Pearson correlation distances. Melon genes were annotated using Gene Ontology (GO) terms [84] before assignment to functional categories by FatiGO analysis [52].

### Microarray validation by real time quantitative reverse transcription PCR (qRT-PCR)

Twelve genes were selected for qRT-PCR analysis to validate the microarray data. The same RNA samples hybridised to the microarray were used for this purpose. First strand cDNA was synthesized from 1 μg of total RNA with an oligo(dT)_20_ primer and a SuperScript™ III Reverse Transcriptase kit (Invitrogen, Carlsbad, CA) according to the manufacturer’s instructions. The resulting cDNA (20 ng per reaction) was analysed by qRT-PCR using a LightCycler® 480 Real-Time PCR System (Roche Applied Science, USA) with SYBR® Green I Mix (Roche Applied Science, USA). Four different reference genes were measured in each sample: *CmEF1α* (elongation factor 1-α), *CmAPT1* (adenine phosphoribosyl transferase), *CmCYP7* (cyclophilin) and *CmACT7* (actin). From analysis of the data with geNorm and BestKeeper software [85, 86], *CmCYP7* was found to be the most stable reference gene and used as the reference in all samples, as in previous experiments [47, 87]. Primers for amplification of target and reference genes were designed with Primer Express® Software v2.0 (Applied Biosystems, Foster City, CA, USA). Primers were based on the 3′-UTR region to ensure target specificity for genes belonging to multi-gene families and are listed in Additional file 4: Table S3. Intra-assay variation was evaluated by performing all amplification reactions in triplicate. A five-point standard curve was constructed for each gene, including *CmCYP7*, from serial 10-fold dilutions of cDNA. Efficiency was calculated from the slope of the linear correlation between Cp (crossing point) values of each dilution and the logarithm of the corresponding amount of RNA, according to the equation *E* = 10^(−1/*slope*)^ − 1 [88]. The amplification protocol consisted of an initial step at 95 °C for 10 min, and 45 cycles of 95 °C for 10 s and 60 °C for 30s. The specificity of the PCR amplification was checked in preliminary experiments by electrophoresis on an Agilent 2100 Bioanalyzer (Agilent Technologies), and routinely in the main experiments by melting curve analysis and agarose gel electrophoresis. Reverse transcriptase minus controls (RT-) and non-template controls (NTC) were included in each plate to assess the presence of genomic contamination (RT-), and primer dimers and/or primer contamination (NTC).

The relative expression of target genes was calculated using Cp values calculated by LC480 software and equation (1):

1 $$ R=\frac{N_{o, target}}{N_{o,HKG}}=\frac{{\left(1+{E}_{HKG}\right)}^{Cp,HKG}}{{\left(1+ Etarget\right)}^{Cp, target}} $$

where HKG is the reference gene (*CmCYP7*). Since the efficiency of amplification (E) was between 0.95-1.05 for all genes, the equation was simplified to equation (2):

2 $$ R=\frac{N_{o, target}}{N_{o,HKG}}={2}^{Cp,HKG-Cp, target} $$

Statistical tests were performed using the SSPS 17 (SSPS Inc., Chicago, III) package. All statistical calculations were performed using ΔCp values, as this parameter followed a normal distribution as assessed by the Kolmogorov-Smirnov test. Data were transformed to a log_2_ scale to make the data comparable with the microarray results. Pearson’s correlation distance was calculated across all four genotypes and fruit developmental stages combined.

### Analysis of differentially expressed genes

Genes related to metabolic or signalling pathways of interest were extracted using annotations in the KEGG gene database (http://www.genome.jp/kegg/) [89] and MapMan ontologies [68, 90]. Genes that were differentially expressed during fruit ripening in Ved and PS were identified by analysis of the microarray data using the Significance Analysis of Microarrays (SAM) algorithm in the TM4 software package [91], and by analysis with the Robin software package [67]. Data were displayed using MapMan [68]. For the assignment of melon genes as homologs or orthologs of previously characterized genes in other plant species, we have used plant phylogeny databases as PhylomeDB (http://phylomedb.org/) and PLAZA v3.0 (http://bioinformatics.psb.ugent.be/plaza/versions/plaza_v3_dicots/).

### Availability of supporting data

The data discussed in this publication have been deposited in NCBI’s Gene Expression Omnibus and are accessible through GEO Series accession number GSE62560 (http://www.ncbi.nlm.nih.gov/geo/query/acc.cgi?acc= GSE62560).

## Additional files

Additional file 1: Figure S1. Expression of ethylene related genes analyzed by qRT-PCR on ethylene-treated fruits. Analysis was done on PS, PI and Dul control and treated fruits. Measurements were performed 24 h after the exogenous ethylene was applied (1000 ppm during 24 h at 20 ºC). a: *CmACS1* (1-aminocyclopropane-1-carboxylate synthase 1; MU51580)*.* b: *CmPG1* (polygalacturonase 1; MU10927). c: *CmACO*1 (1-aminocyclopropane-1-carboxylate oxidase 1; cCL451Contig1). d: *CmXTH1* (xyloglucan endotransglucosylase/hydrolase 1; cCL6092Contig1). e: *CmETR1* (ethylene receptor 1; cCI_54-H09-M13R). Black boxes indicate gene expression in ethylene-treated fruits. White boxes indicate the expression in control fruits (without external ethylene treatment). Additional file 2: Table S1. One-way ANOVA test on the sugar data in Figure 2 to provide statistical analysis. The file shows the P-values for each pairwise comparison of time points for each sugar end melon genotype. P-values with values P < 0.05, P < 0.01 and P < 0.001 are highlighted. Additional file 3: Table S2. List of unigenes represented in the microarray with the corresponding gene code from the genome sequencing [48] and the ICuGI code (http://www.icugi.org). The average expression value of each gene in the microarray is also given for all genotypes during the four ripening stages. The annotation of each gene is also shown as described in each source (genome, Melogen or ICuGI). Additional file 4: Table S3. Primer sequences of the genes used for the validation of the microarray expression data with qRT-PCR. Additional file 5: Figure S2. Comparison of microarray and qRT-PCR expression of *CmACO1* (1-aminocyclopropane-1-carboxylate oxidase-1)*.* a: Log2 fold change relative to 15 DAP for each genotype from array *CmACO1* gene expression values. b: Log2 fold change relative to 15 DAP from qRT-PCR *CmACO1* gene expression values. Additional file 6: Table S4. List of the differential expressed genes in at least one genotype, during fruit development. Differentially expressed genes for each genotype are also listed in different Excel spreadsheets. Additional file 7: Figure S3. MaSigPro analysis of the differentially expressed genes in each variety during “TIME”, showing the distribution of the corresponding GO terms. PS (‘Piel de Sapo’), PI (PI 161375), Ved (‘Védrantais’) and Dul (‘Dulce’). Differentially expressed genes were 2186 (PS), 3808 (PI), 6670 (Ved) and 3597 (Dul). Additional file 8: Table S5. Gene Ontology terms (GO terms) of the nine different clusters obtained according to the differentially expressed gene pattern. Genes belonging to each cluster are listed in contiguous spreadsheets. % vs. array means the representation of the GO term in relation to the complete array. Significant values are those higher than 50 %. No significant GO terms were found in clusters C8 and C9. Additional file 9: Table S6. Members of the *CmACS* and *CmACO* families (1-aminocyclopropane-1-carboxylate synthase and 1-aminocyclopropane-1-carboxylate oxidase, respectively) extracted from the melon genome sequence. a: gene not present in the microarray, b: gene not present in the ICuGI database and c: gene not present in Genbank. Additional file 10: Table S7. Expression values of genes related to ethylene biosynthesis, perception and signalling (spreadsheet 1) and carotenoid biosynthesis (spreadsheet 2). Genes highlighted in yellow are those discussed in the main text. Additional file 11: Table S8. MapMan analysis of Ved and PS at harvest stage (spreadsheet 1). Ethylene and carotenoid biosynthesis, transcription factors, sugar and cell wall related genes were analyzed. De-regulated genes were also detected by running the SAM tool (spreadsheet 2). Genes more expressed in Ved have positive values and genes more expressed in PS have negative values. Genes highlighted in yellow are those discussed in the main text.

## Abbreviations

DAP: Days after pollination
PI: Accession PI 161375
PS: cv. Piel de sapo T111
qRT-PCR: quantitative reverse-transcription polymerase chain reaction
Ved: cv. Védrantais
Dul: cv. Dulce
