# Supplementary material for: Comparative transcriptional profiling analysis of developing melon (Cucumis melo L.) fruit from climacteric and non-climacteric varieties
Source: BMC Genomics. 2015 Jun 9;16(1):440. doi: 10.1186/s12864-015-1649-3 (PMC4460886; doi:10.1186/s12864-015-1649-3)
Supplement: Additional file 1: Figure S1. — Expression of ethylene related genes analyzed by qRT-PCR on ethylene-treated fruits. Analysis was done on PS, PI and Dul control and treated fruits. Measurements were performed 24 h after the exogenous ethylene was applied (1000 ppm during 24 h at 20 ºC). a: CmACS1 (1-aminocyclopropane-1-carboxylate synthase 1; MU51580). b: CmPG1 (polygalacturonase 1; MU10927). c: CmACO1 (1-aminocyclopropane-1-carboxylate oxidase 1; cCL451Contig1). d: CmXTH1 (xyloglucan endotransglucosylase/hydrolase 1; cCL6092Contig1). e: CmETR1 (ethylene receptor 1; cCI_54-H09-M13R). Black boxes indicate gene expression in ethylene-treated fruits. White boxes indicate the expression in control fruits (without external ethylene treatment). [file 12864_2015_1649_MOESM1_ESM.pptx]

## Slide 1
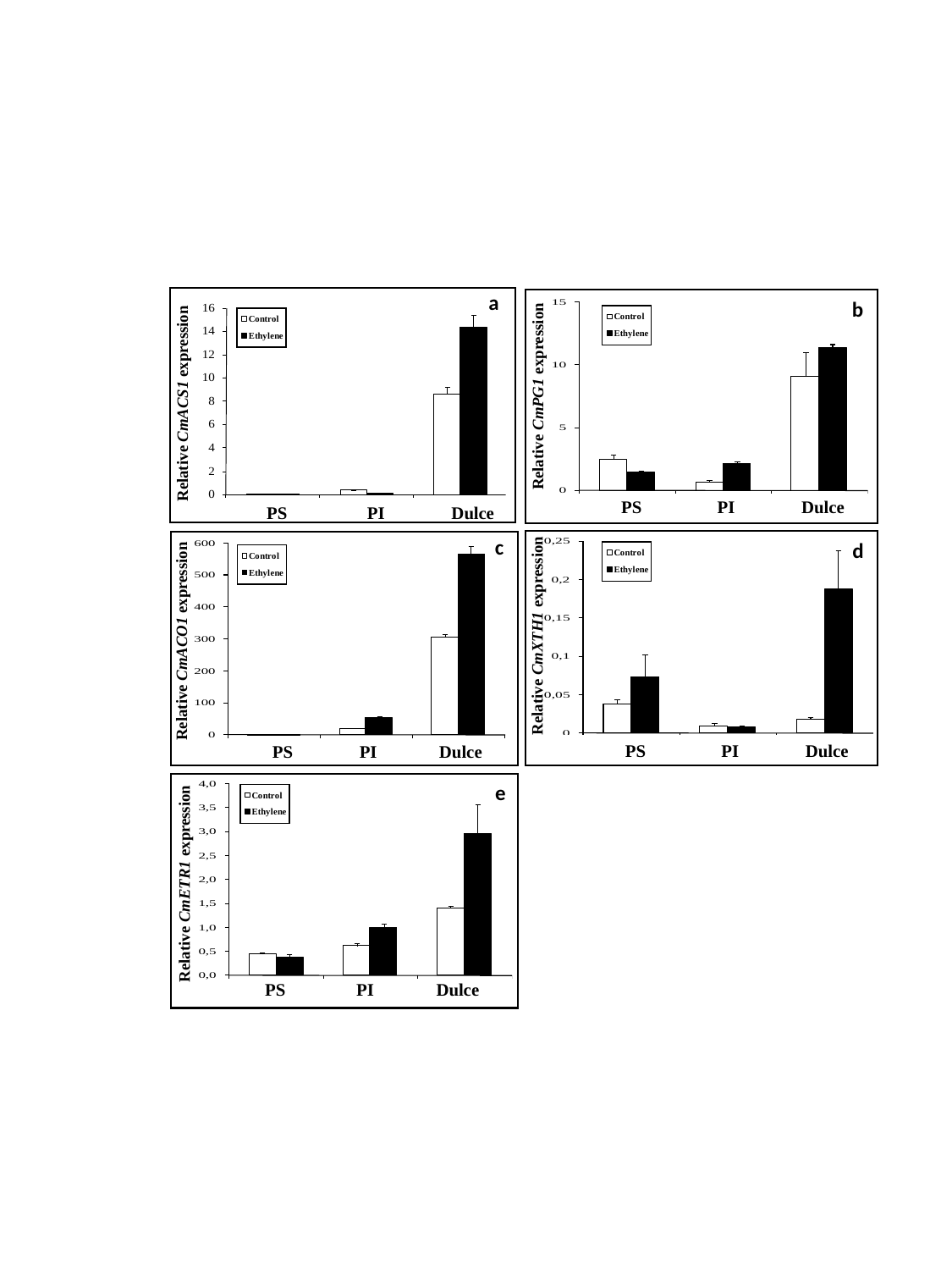

a
Relative CmACS1 expression
PS PI Dulce
c
Relative CmACO1 expression
PS PI Dulce
e
Relative CmETR1 expression
PS PI Dulce
b
Relative CmPG1 expression
PS PI Dulce
d
Relative CmXTH1 expression
 PS PI Dulce
