# Supplementary figures and images for: Comparative transcriptional profiling analysis of developing melon (Cucumis melo L.) fruit from climacteric and non-climacteric varieties
Source: BMC Genomics. 2015 Jun 9;16(1):440. doi: 10.1186/s12864-015-1649-3 (PMC4460886; doi:10.1186/s12864-015-1649-3)

## Slide 1
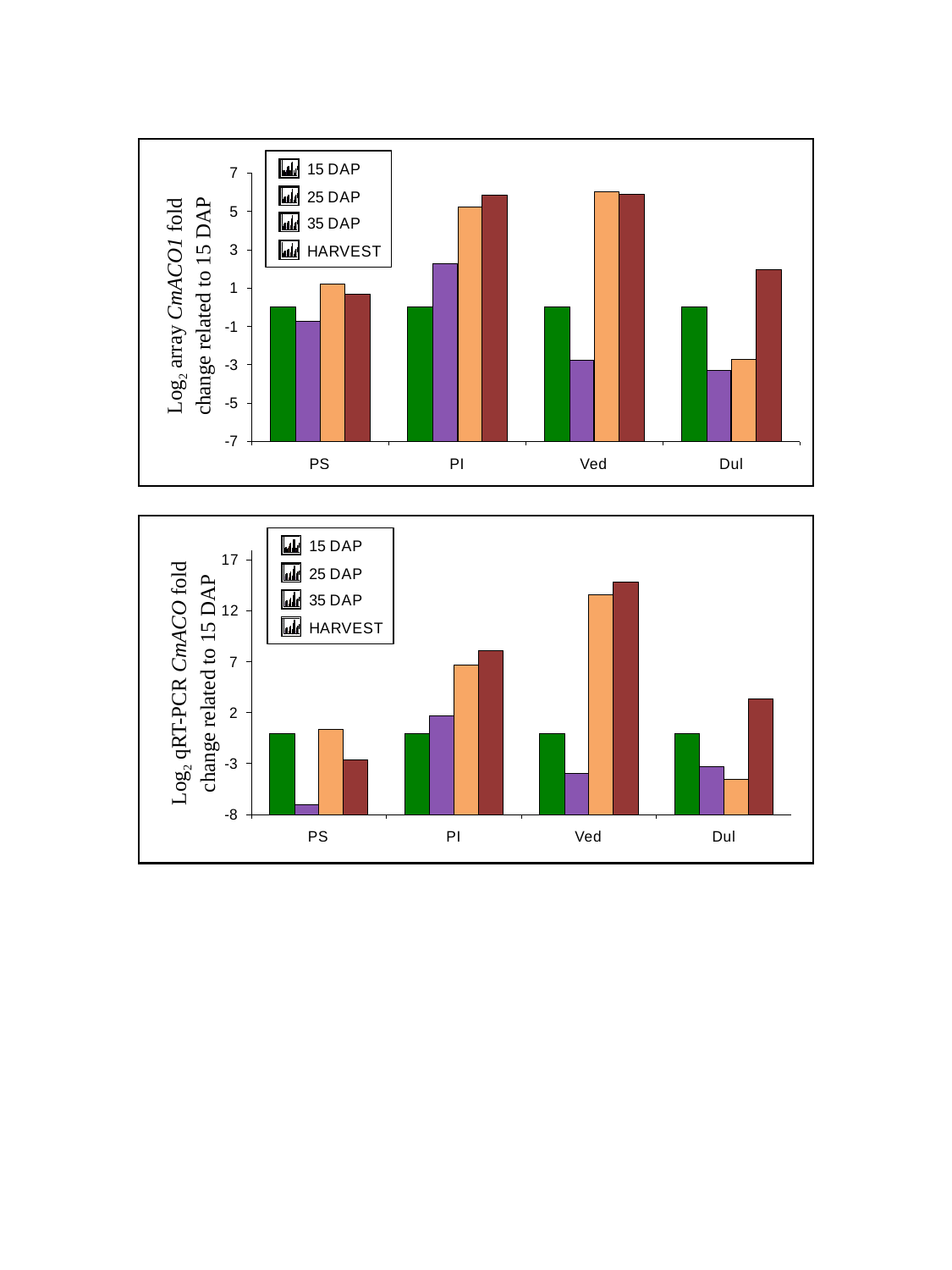

Log2 array CmACO1 fold change related to 15 DAP
Log2 qRT-PCR CmACO fold change related to 15 DAP

Supplement: Additional file 5: Figure S2. — Comparison of microarray and qRT-PCR expression of CmACO1 (1-aminocyclopropane-1-carboxylate oxidase-1). a: Log2 fold change relative to 15 DAP for each genotype from array CmACO1 gene expression values. b: Log2 fold change relative to 15 DAP from qRT-PCR CmACO1 gene expression values. [file 12864_2015_1649_MOESM5_ESM.ppt]

## Slide 1
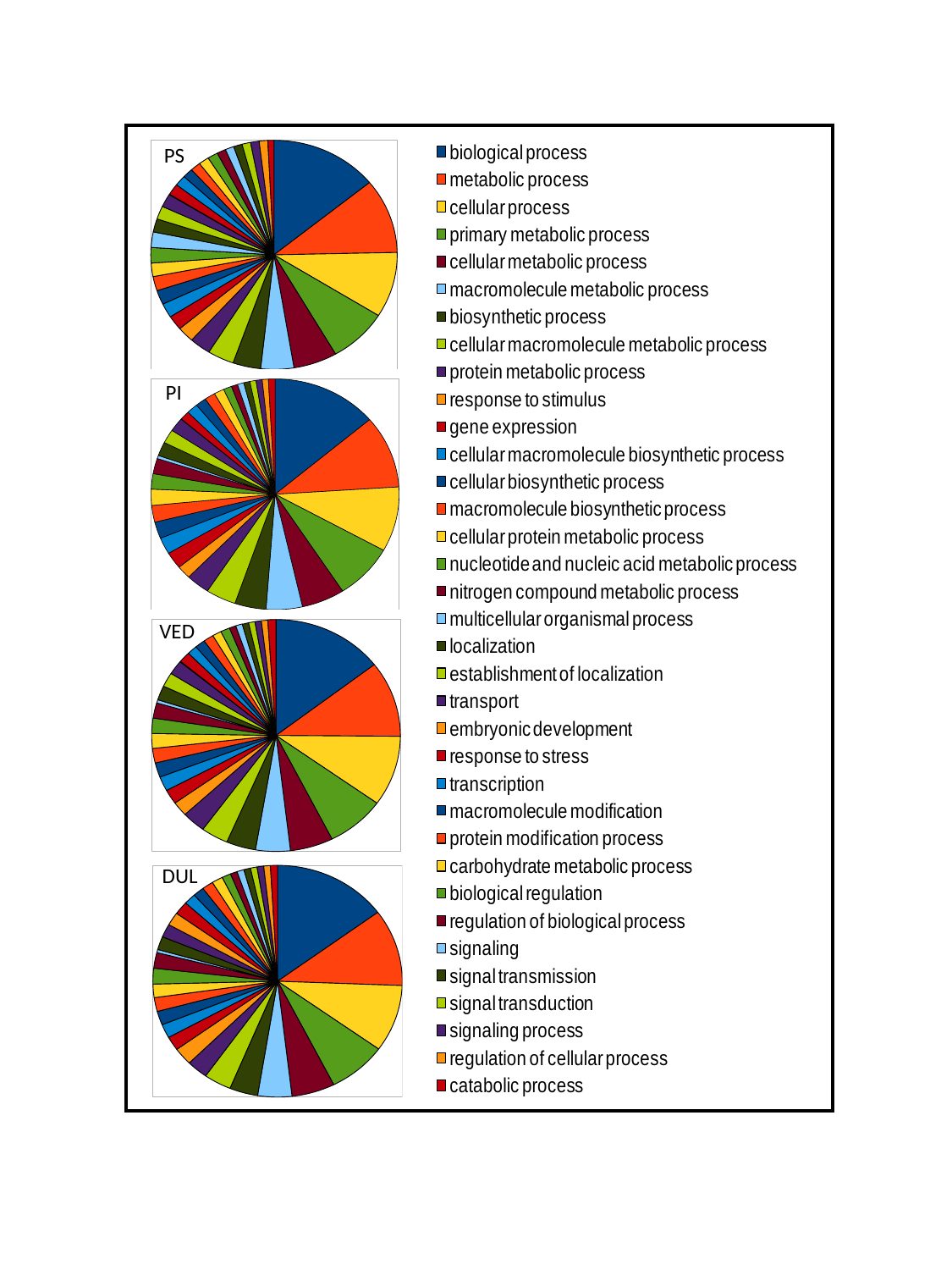

PS
PI
VED
DUL

Supplement: Additional file 7: Figure S3. — MaSigPro analysis of the differentially expressed genes in each variety during “TIME”, showing the distribution of the corresponding GO terms. PS (‘Piel de Sapo’), PI (PI 161375), Ved (‘Védrantais’) and Dul (‘Dulce’). Differentially expressed genes were 2186 (PS), 3808 (PI), 6670 (Ved) and 3597 (Dul). [file 12864_2015_1649_MOESM7_ESM.ppt]
